# Supplementary material for: Association between oral microbiome and five types of respiratory infections: a two-sample Mendelian randomization study in east Asian population
Source: Front Microbiol. 2024 Apr 10;15:1392473. doi: 10.3389/fmicb.2024.1392473 (PMC11039966; doi:10.3389/fmicb.2024.1392473)
Supplement: Supplementary file 1 [file Data_Sheet_1.zip › Supplementary Materials/MR plots of saliva/Tonsillitis/s__Pauljensenia_sp000466265_mgs_216/forest.pdf]

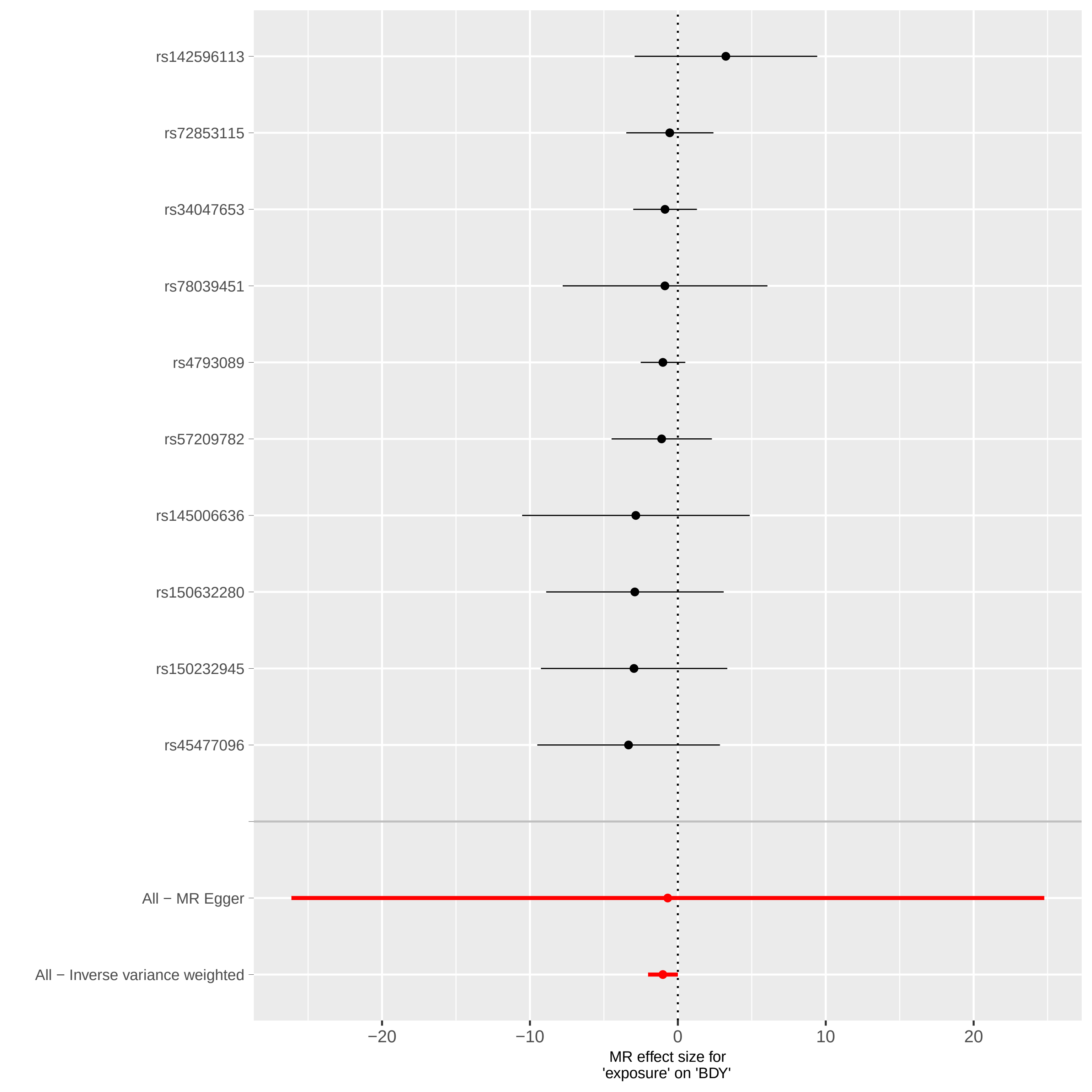

rs142596113
rs72853115
rs34047653
rs78039451
rs4793089
rs57209782
rs145006636
rs150632280
rs150232945
rs45477096
All − MR Egger
All − Inverse variance weighted
−20
−10
0
10
20
MR effect size for
'exposure' on 'BDY'
